# Raymond Tallis

**DOI:** 10.1192/bjb.2021.65

**Published:** 2021-10

**Authors:** Abdi Sanati

**Figure:**
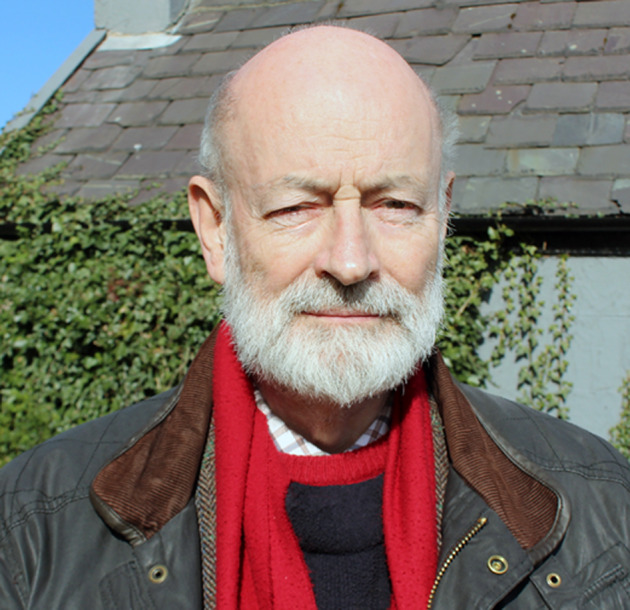

Professor Raymond Tallis is one of the most eminent polymaths in the UK. He is an Emeritus Professor of Geriatric Medicine, poet, novelist, philosopher and cultural critic. He has published numerous books and articles in different fields, including his regular contribution to *Philosophy Now* under the title of Tallis in Wonderland. I first came across his work in his book *Aping Mankind*.^1^ Professor Tallis is politically active and his co-edited book *NHS SOS*,^2^ written at the time of Andrew Lansley's reorganisation of the NHS, continues to be relevant. I met Professor Tallis virtually in the middle of the lockdown.

**Professor Tallis, thank you for your time. In the Royal College of Psychiatrists, we recently had a kind of surge in neuroscience and I remember 10 years ago, in *Aping Mankind*, you wrote of ‘neuromania’. I wonder if you could elaborate for the readers.**

Of course. First of all, I am a great admirer of neuroscience: it is perhaps the greatest cognitive achievement of humanity, because it brings together so many sciences in an area that has such importance. All my own research has been within clinical neuroscience, stroke and epilepsy being my major areas of research. I therefore have no problem with neuroscience. My target is neuromania. What is the difference between neuroscience and neuromania? Neuroscience does acknowledge that the brain is a necessary condition of normal consciousness and behaviour. If you chop my head off my IQ falls precipitously, probably down to zero, I guess! In addition, we are very familiar with the effects of damage: there is often very precise correlation between severe damage to the brain and deficits that follow. There are lower-level deficits in sensation and motor activity and higher-level deficits in cognitive function. So that is neuroscience: it acknowledges the brain as a necessary condition of normal human consciousness, behaviour, awareness and so on. Neuromania, however, claims that the brain is not merely a necessary condition, but a sufficient condition of these things. All that we are as persons can be translated into neural activity.

It might be helpful for your readers to clarify the difference between necessary and sufficient conditions. Let me give you an example. To be knocked down by a bus outside the Royal College of Psychiatrists, it is necessary that I should be outside the Royal College of Psychiatrists. I am very pleased to say that it is not a sufficient condition. Otherwise, everybody outside the Royal College of Psychiatrists would be knocked down by a bus. According to those who embrace neuromania, the stand-alone brain is the sufficient condition for human consciousness, the person and so on. Neuroscience tells us the brain is merely a necessary condition.

**One thing we have witnessed is a huge surge in imaging studies, fMRI studies in particular. And they want to say that, for instance, this is what love looks like. Or in terms of psychiatry, this is what, for instance, depression looks like. It is kind of reductionism.**

It is, and of course, imaging is particularly seductive. Someone once described the fMRI scan as a fast-acting solvent of the critical faculty. So, if you see an image on a screen you start believing that you are gaining direct access to the truth about our humanity. You mentioned love and it reminds me of a study by Semir Zeki and his colleagues that led them to identify the experience of romantic love with activity in certain parts of the nervous system. The experimental design was, to put it mildly, rather crude. They compared the responses of the brain, recorded by fMRI, when individuals were exposed to pictures of partners with whom they were deeply in love with the responses to pictures of friends. By this means, they claimed to identify brain activity corresponding to romantic love. But of course, that's nonsense. Being in love is like a response to a stimulus. It's not even a continuous condition like being a bit chilly or being a bit cross. It is a very complicated business. It has narrative and cultural contents. And it is associated with all sorts of things, like deciding not to talk to somebody because you are very cross with them. All of those things are incredibly complex, and remote from responding to an external stimulus such as a picture. But Zeki et al's experiment is a good example of how neuromania tries to reduce the life of a person to a succession of cerebral discharges.

**I have been thinking about depression because it has many different dimensions. The experience of someone who is depressed is very complex. Trying to reduce it to certain kinds of activities of the brain won't work. They are very different.**

Depression has a major narrative content in it. It reflects your interpretation of the world and of your place in it. It encompasses what you are telling yourself about yourself, what you tell yourself about others and what you tell yourself about the world. These are not going to be connected in any direct or simple way to alterations in neural activity explained by depletions in particular neurotransmitter pathways, even though this may have a causal role.

**Another issue that we have in psychiatry and we deal with it a lot, especially in forensic psychiatry, is the issue of free will and responsibility. I have talked to many people and they said, well, neuroscience has solved the problem; there is no such thing as free will. What is your opinion on that?**

You will not be surprised to know what my opinion is. I have just finished a book on free will.^3^ I devoted a section to so-called neurodeterminism and to the neurological experiments that are supposed to demonstrate that we do not have free will.

It is worthwhile looking at the most famous ones conducted by Benjamin Libet, 30 or more years ago, but repeated many times since in different forms. What Libet did was to ask study participants to make a very simple movement, just basically flexing their wrist or flexing their finger, but in their own time, so it was a genuinely voluntary movement. He also asked them to do something else: to time the moment when they felt the *urge* to make the movement by noting the position of a spot moving on a clock face on a screen. At the same time, Libet recorded the so-called readiness potential, which is supposed to be a marker in the prefrontal cortex of preparedness to make a movement. What he found was that the readiness potential preceded the urge to make the move. It was as if the brain were getting geared up to make a move, even before the person was aware of the urge to do so. And it has been concluded from this that the brain, not the conscious person, was calling the shots. The decision to move had already been made before the person had experienced the urge to do so. The readiness potential was ahead by only 0.3 s and there are many reasons for thinking that this might not be significant. But then John-Dylan Haynes and colleagues performed similar experiments around 20 years later. Instead of relying on EEG he employed fMRI. He found that the interval between seemingly relevant neural activity and the urge to make a movement (timed by noting letters passing down a screen) was between something like 5 and 10 s and that looked really serious. In Haynes's study, participants were asked to choose to move either their right hand or their left hand to press a button. That study seemed to indicate that the brain was getting all geared up to make a movement well before the person had made a decision to move.

There are two types of fundamental problem with these studies. There are the methodological problems. But more interesting are the philosophical problems arising from a complete misunderstanding of the nature of human action.

Let me first talk about the methodological problems. There are problems about timing an urge. How long does it take you, as it were, to register an urge to enable you to time it? There was also some question about the nature of the readiness potential. It may well be the case that when I decide to make my movement, it is because I feel like I'm taking advantage of the readiness potential: I am, as it were, sort of surfing a wave. And there are many other methodological problems.

But there are more important problems due to a misunderstanding of the nature of action. Think about the experiment from the point of view of the participant taking part in it. Mrs Smith decides that she is going to volunteer for Professor Libet's experiment. She rings up the laboratory, having seen the advert in the newspaper, makes an appointment for 2 weeks' time, sets her alarm the night before to make sure she does not oversleep and upset Professor Libet. She sets out in the car, having made arrangements for the children to be looked after. She arrives at the research centre and has a blazing row with somebody in the car park because they have taken the parking spot that has been set aside for her. She eventually finds her way to the laboratory. She sits down and listens to Professor Libet, and is persuaded that it is a safe experiment to take part in, despite all the people in white coats and all the intimidating machinery around her and the electrodes placed on her head. She pays attention so that she understands what she has to do. This background makes it evident that her action is not just moving a finger. It's ‘taking part in Professor Libet's experiment’, and clearly that is not something that is the product of an atomic urge, a bit of willing causing a little movement. Taking part in the experiment is actually much more reflective of the complexity evident in even the most ordinary human actions. Most striking is the temporal depth of the action – setting the alarm to get to the lab on time and so on. And then there's the question of motivation. Perhaps Mrs Smith decided she wanted to take part in the experiment so those clever scientists might find something about brain entity, which might help a little boy next door who has brain injury problems.

It will be evident from this that most of what voluntary human action is about cannot possibly be captured in experiments like Libet's. And what about the other participants in the experiments – Professor Libet and Professor Haynes? They too are agents, and they probably had to apply for a grant a couple of years before the experiments. They had to work out the experiments and undergo training in order to understand how to investigate the questions they wanted to explore. They had to perform these experiments, and then they had to write all this stuff up.

The point is this: human agency is very complex, and it is not made of little atomic urges and twitches. That is why arguments for determinism based on these kinds of so-called empirical experiments don't impress me the slightest bit. I think they have nothing to say about whether we do or do not have free will.

**I assume that Libet and Haynes were not dualists, or perhaps to put it better, substance dualists. They are more physicalists in terms of philosophy of mind. What would they expect to see? Did they expect the person to decide before their brain? In that case, where would be the locus of decision? I wonder what would they expect to see in the case of free will? They said because the brain activity happened before the desire there's no free will. But what about the other way around? Would that be satisfying, if the urge happens before the brain activity? What would the source of the urge be?**

It has been said that Libet was a dualist and he somehow thought you and your urges are separate and independent from the things you do. I do not think we can ascribe a clear metaphysics to Libet, apart from a commitment to identifying ourselves with neural activity. And once you identify yourself with neural activity, you identify it with physical events, which are necessarily causally stitched into the flow of other physical events. If I say that my free will is to be identified with neural activity, then of course I have already given up on the possibility of free will.

There are many reasons why we cannot identify exercise of free will or normal agency with neural activity. First, when I'm performing an action, that action is associated with an intention, an intention that is not localised in this moment in time. It reaches back to a past that makes sense of my intention and towards something that doesn't yet exist, a future, which also makes sense of my intention. And without that temporal depth, which doesn't exist in the material world, there will be no such thing as meaningful intentional action.

**There is another topic I wanted to discuss. I think in 2012, you edited a book with Jacky Davis called *NHS SOS*.** ^2^ **How do you see the progress and development of the NHS since then?**

I have spent an awful lot of time in the intervening 8 or 9 years, marching, waving banners, writing to MPs and so on, though not in the past year of course, because of COVID. And it seems to me that we've gradually moved to a much more privatised NHS. But it's been done much more subtly. After the Lansley Bill [the Health and Social Care Bill of 2011], and there was an enormous amount of anger about it, they decided they needed to be more subtle. But it's interesting how in the COVID crisis, we've had a brilliant demonstration of how the NHS works and how privatisation and outsourcing doesn't. If you want to waste £37 billion, give it to the private sector – Serco and others – and get them to run Test and Trace. If you want to actually have something that's really successful, get it run by the NHS – that's why the vaccination scheme, which has been disseminated through the vascular tree of the NHS, has worked so well. So, we've had a very interesting comparison between things that are outsourced to the private sector, incredibly expensive, totally wasteful, and things that have remained within the NHS and have been very successful.

**Yes, we have this new White Paper** ^4^ **that wants to integrate cooperation instead of competition. I might well be wrong but I don't think competition worked.**

You're absolutely right. The White Paper at first sight looks really good. They said let's say farewell to obligatory competitive external tendering. Great. Let's say farewell to internal market. Great. Let's integrate health and social care. Great. But… but… but. We need to be very careful for two reasons. First, when you integrate health and social care you're integrating something which is free at the point of need and still mainly publicly provided (health) with something that is overwhelmingly privately provided and is means tested (social care). But when you bring those two together there's the danger that it could go either way: to an entirely publicly provided, free at the point of need service; or to entirely privately provided services subjected to means testing. The second reason relates to the question of who is actually responsible for the ‘integrated care system’. It is not impossible that the system could be run from outside the NHS, even from an American firm. We can see how real this possibility is by the gigantic privatisation initiative that is the disastrous Test and Trace. And the recent takeover of nearly 50 general practices in London by Centene in the past few weeks.^5^ So, whereas I like the overall principles in the White Paper, I worry that it could be the opportunity for the biggest privatisation of all.

**There is a risk of that. And one thing I have observed is the amount of bureaucracy. That is exponentially growing. People talk about being more lean but I think this is only when they are discussing finances – not many are thinking lean when it comes to paperwork.**

If you compare the percentage of funding that is spent on administration with that spent on clinical care, it is much greater in privatised health services than in the NHS as it was. We are still a long way behind, for example, the USA in terms of the proportion of our funding that is spent on administration, but the internal market and the external market with compulsory competitive tendering has closed the gap. So, one would hope that the new White Paper^4^ would genuinely liberate funds, taking them away from bureaucracy and administration and bring them back to the front line of clinical care. But there's no doubt about it – if we have a systemic privatisation, which I feel we could have with an integrated care system, as opposed to episodic privatisation, as we have at the moment, we will spend yet more on administration – as well as converting more of taxpayer's money into profits to be hidden in off-shore tax havens. And also, it'll be more expensive. I mean, think of the States – they spend 18% of their GDP on healthcare, and they have a worse system. Many US citizens have only minimal healthcare coverage.

**Thank you very much for your time.**

## References

[1] Tallis R. Aping Mankind: Neuromania, Darwinitis and the Misrepresentation of Humanity. Routledge, 2011.

[2] Davis J, Tallis R (eds). NHS SOS: How the NHS Was Betrayed and How We Can Save It. Oneworld Publications, 2013.

[3] Tallis R. Freedom: An Impossible Reality. Agenda Publishing, 2021, in press.

[4] Department of Health & Social Care. Policy Paper: Working together to Improve Health and Social Care for All. GOV.UK, 2021 (https://www.gov.uk/government/publications/working-together-to-improve-health-and-social-care-for-all [cited 13 Apr 2021]).

[5] Iacobucci G. Subsidiary of US healthcare firm will run more than 50 GP practices after takeover deal. BMJ 2021; 372: n519. 10.1136/bmj.n519 33619035

